# Pediatric Dentists’ Service Provisions in South-East Europe during the First Wave of COVID-19 Epidemic: Lessons Learned about Preventive Measures and Personal Protective Equipment Use

**DOI:** 10.3390/ijerph182211795

**Published:** 2021-11-10

**Authors:** Ana Vuković, Stefan Mandić-Rajčević, Ruxandra Sava-Rosianu, Marcela D Betancourt, Edit Xhajanka, Neada Hysenaj, Elmedin Bajric, Amila Zukanović, Vrassidas Philippides, Marios Zosimas, Maroufidis Nikolaos, Zoran Vlahović, Marijan Denkovski, Tamara Peric, Dejan Markovic, Guglielmo Campus

**Affiliations:** 1 Department of Pediatric and Preventive Dentistry, School of Dental Medicine, University of Belgrade, 11000 Belgrade, Serbia; ana.vukovic@stomf.bg.ac.rs (A.V.); tamara.peric@stomf.bg.ac.rs (T.P.); dejan.markovic@stomf.bg.ac.rs (D.M.); 2 School of Public Health and Health Management, Institute of Social Medicine, Faculty of Medicine, University of Belgrade, 11000 Belgrade, Serbia; 3 Department of Health Sciences, University of Milan, 20122 Milan, Italy; 4 Department of Preventive, Community Dentistry and Oral Health, Faculty of Dentistry, University of Medicine and Pharmacy “Victor Babes”, 300041 Timisoara, Romania; sava-rosianu.ruxandra@umft.ro; 5 Department of Pediatric and Preventive Dentistry, Dental Clinic, University of Bern, 3012 Bern, Switzerland; marcela.betancourt@zmk.unibe.ch (M.D.B.); guglielmo.campus@zmk.unibe.ch (G.C.); 6 Faculty of Dental Medicine, University of Medicine, 1005 Tirana, Albania; edit.xhajanka@umed.edu.al (E.X.); neada.hysenaj@umed.edu.al (N.H.); 7 Department for Preventive and Paediatric Dentistry, Faculty of Dentistry, Sarajevo University, 71000 Sarajevo, Bosnia and Herzegovina; ebajric@sf.unsa.ba (E.B.); azukanovic@sf.unsa.ba (A.Z.); 8 Cyprus Dental Association, Nicosia 2414, Cyprus; phivra@cytanet.com.cy (V.P.); mzosimas@yahoo.com (M.Z.); 9 Hellenic Dental Association, 10678 Athens, Greece; nikmaroufidis@yahoo.gr; 10 Department of Dentistry, Faculty of Medicine, University of Pristina, 38220 Kosovska Mitrovica, Serbia; zoran.vlahovic@med.pr.ac.rs; 11 Macedonian Dental Chamber, 1300 Kumanovo, North Macedonia; marijan_denkovski@yahoo.com

**Keywords:** COVID-19, SARS-CoV-2, dentistry, pediatric dentistry, infection control, public health dentistry

## Abstract

Introduction: Having in mind the importance of providing continuous pediatric dental services during the COVID-19 pandemic and the fact that children have similar viral loads to adults, the potential to spread the virus to others, and with variable clinical presentation of COVID-19 infection, this study aimed to analyze the impact of COVID-19 outbreak on pediatric dentistry service provision, risks, and preventive measures before and during dental treatment. Method: Structured and closed epidemiological cross-sectional survey involved seven Southeastern European countries. The questionnaire was developed using the modified Delphi method, pretested, and tested in North Italy during April 2020. The sample consisted of licensed dental professionals reached via national dental chambers and social media using the best strategies according to the national setting. Results: A total of 3227 dentists participated in the survey, and we included 643 specialists in this study—among them, 164 were pediatric dentists. Most pediatric dentists worked in the public sector (61.0%) and provided emergency (64.6%) and routine dental treatment (18.3%) during the outbreak. One-third of pediatric dentists were COVID-19 tested, statistically significantly more than other specialties, and 3.0% tested COVID-19 positive. In addition, significantly more pediatric dentists (13.4%) reported the presence of at least one symptom related to COVID-19 compared to other specialists (6.1%). None of the pediatric dentists reported PPE shortage. However, 26.2% of all specialists stated that they lacked clear step by step professional guidance in a national language. Similarly, in both groups, around 10% of specialists attended education on coronavirus. Conclusions: Considering that most pediatric dentists provided dental treatment during lockdown in their countries in public health centers and that they will continue to work during pandemic, our results suggest that pediatric dentists might be at higher risk of COVID-19 infection. Further research should focus on finding better ways to promote and adapt preventive, protective measures and PPE in the pediatric dental setting to be behaviorally acceptable. Moreover, additional efforts should be invested in dental education regarding COVID-19 in the mother tongue.

## 1. Introduction

Children and adolescents usually present with milder COVID-19 compared to adults [1,2]. Luckily, most children, especially the youngest, usually have asymptomatic infection or not as many symptoms as adults do have, mainly involving only mild gastrointestinal inconveniences [3]. While SARS-CoV2 testing initially focused on the diagnosis and treatment of symptomatic patients, this effort has expanded to include the surveillance of asymptomatic patients due to reports of thousands of healthcare workers infected probably due to transmission from asymptomatic patients despite adherence to infection control measures [4]. In addition, data on COVID-19 prevalence in children might be underestimated and should be cautiously interpreted due to the lack of widespread testing and the prioritization of testing for adults and those with severe illness, especially in low-resource settings [5].

New evidence suggests that children contribute to community transmission due to undetected and mild cases [6]. Special attention should be focused on COVID-19 infection prevention and protection of pediatric dentists—oral health professionals providing face-to-face procedures to children, creating bioaerosol and spatter, and exposing themselves to blood, saliva, and other body fluids. Above all, the amount of aerosols and droplets produced by an uncooperative child during chairside dental treatment multiplies the risk for infection transmission [7].

Considering that oral health was marginalized from general health policies even in the pre-COVID era [8], raising awareness on the importance of safely continuing pediatric dental practice through pandemic times is crucial. There has been an incredible surge in knowledge during the last year; besides the availability of recommendations and guidelines, research articles exploring the prevalence of COVID-19 and pediatric dentistry risks are scarce. The authors firmly believe that more knowledge on this matter would empower implementing strategies for better infection prevention and control, keeping all stakeholders safe—pediatric dentists, their vulnerable little patients, and their families.

Taking into account the hypothesis that pediatric dentists may represent a particularly high-risk group among oral health professionals during the pandemic, an epidemiological survey was designed, planned, and carried out, aiming to describe and analyze the impact of COVID-19 outbreak on pediatric dentistry service provision and assess risks among pediatric dentists compared to other oral health specialists in Southeastern Europe.

## 2. Materials and Methods

### 2.1. Study Design

Data presented in this survey are part of the 2020 International Collaborative COVID-19 Disease Study, with a previously registered protocol at the World Pandemic Research Network (WPRN) WPRN-486352 [9]. The central management team of COVIDental Collaboration Group invited 36 collaborating research groups worldwide [10]. According to the protocol designed by the central coordinator (GC) and his team, our research team sent 13 invites to the collaborators from other countries geographically corresponding to the Southeastern Europe. Collaborators from eight countries replied positively and were involved in this survey (Table 1). Since dentists in Romania (*n* = 336) did not declare their type of specialty, they were excluded from the final analysis in this manuscript.

The survey was approved by the Ethical Committees of the appropriate institutions in each participating country according to the national regulations: University of Medicine of Tirana in Albania, Dental Chamber of Montenegro, Macedonian Dental Chamber, University of Medicine and Pharmacy Timisoara in Romania, and School of Dental Medicine University of Belgrade in Serbia. In Bosnia and Herzegovina, Cyprus, and Greece, ethical approval for anonymous questionnaires was not required.

The structured and closed cross-sectional survey was developed using a previously standardized questionnaire available as supplement material in a previously published paper by the wider international team [10]. The questionnaire was designed by modified Delphi method and involved the following domains: (1) general data (personal, demographic, and working status); (2) data regarding COVID-19 infection rate and symptoms; and (3) use of preventive and prophylactic measures, PPE, and risk perception [9,10]. The study protocol was previously pretested on 12 dentists; only items showing Intra-class Correlation Coefficient higher than 0.80 were further tested and evaluated among 3599 dentists in North Italy in April 2020 [11]. The questionnaire was translated, culturally adapted, semantically adapted if necessary, and piloted in each country. The same core questionnaire was used in all countries.

### 2.2. Settings

Researchers from eight Southeastern European countries agreed to participate in the present survey. All the countries involved in the study had a state of emergency in the beginning of the COVID-19 pandemic in similar periods during spring of 2020, as displayed in Table 1. In all countries, private dental offices were closed during lockdowns, with National Dental Chambers recommending only emergency dental treatment in public dental institutions or with special approvals in (voluntarily) dedicated private offices.

The survey was run during June and July 2020 after the end of emergency state situations, and the questions were related to the period during the state of the emergency. Data collection was performed using LimeSurvey’s open source tool [12] with all national questionnaires and survey/question logic coded into the software. The LimeSurvey software and the data bank were housed on the secure servers of the Faculty of Medicine, University of Belgrade. Individual data collection link was available for each language/country. The invitation to complete the survey using the link was sent through National Dental Chamber resources, such as mailing lists and official web pages and social media (Facebook, WhatsApp, and Viber groups for sending reminders). Each country’s social media promotional video was made in the national language using the tutorial version of VideoScribe (created by Sparkol).

### 2.3. Participants

The study sample involved licensed dental professionals working in each national health system, private or public, including general or specialist dentists. Considering the total population of registered dentists of 24,000 in the Southeastern Europe region, the 95% confidence level, and the margin of error of 2%, the calculated sample size was 2100 participants. For the subgroup of pediatric dentists and to estimate the prevalence of SARS-CoV-2 based on the expected prevalence of SARS-CoV-2-positive dentists of 10% (±5%), according to previous reports in frontline and non-front line healthcare workers and a confidence level of 0.95, the adequate proposed sample was 139 dentists. The total number of working dental professionals was identified using National Dental Chamber data recourses. According to the previously published protocol, at least 5–20% of the total dentist population in each country was invited to participate; in Southeastern Europe, all registered dentists were invited using National Dental Chambers’ resources. The aim was to reach a minimum 5% proportion of all registered dentists as the final sample [9].

### 2.4. Outcome and Independent Variables

The outcome (dependent) variables were (1) the presence of SARS-CoV-2 infection symptoms and (2) the frequency of COVID-19-positive tests among pediatric dentists. Prevalence of SARS-CoV-2 infection symptoms was described as the occurrence of at least one or more self-reported, specific, or nonspecific COVID-19 symptoms, such as fever (>37.5 °C), cough, fatigue, difficulty breathing, nasal congestion, headache, running nose, sore throat, generalized pain, diarrhea, loss of smell, loss of taste, and conjunctivitis [10]. The frequency of COVID-19-positive tests among pediatric dentists was calculated using data when responders answered they tested positive during the first wave of the pandemic, or they were hospitalized due to COVID-19 infection.

The following independent variables were involved in the analysis: type of specialty, type of practice, working regime during the state of emergency, and use of precautionary measures and PPE in the dental office [11]. All responders were categorized into two major groups: pediatric dentists and other specialists.

### 2.5. Statistical Methods

Data were analyzed using SPSS 20.0 (IBM Corp. Released 2011. IBM SPSS Statistics for Windows, Version 20.0. Armonk, NY, USA: IBM Corp.) and R 3.4.2 [13]. Results are presented as count (%), means ± standard deviation, or median (25th–75th Percentile) depending on data type and distribution. Continuous variables were compared using parametric (*t*-test), and the independence of two variables in a contingency table was tested using the chi-square test. Odds ratio of having experienced symptoms depending on the type of specialty was determined using univariate logistic regression. *p*-Values less than 0.05 were considered significant.

## 3. Results

A total of 3227 dentists from eight Southeastern European countries filled in the questionnaire, and 979 (30.3%) declared themselves as specialists. However, since specialty dentists in Romania (*n* = 336) did not declare their specialty, they were excluded from the analysis, as it was not possible to differentiate pediatric dentists among them. Therefore, a total of 643 oral health specialists were included in the analysis. More than one quarter 25.5% (*n* = 164) of all specialists from the sample were pediatric dentists. There was no statistically significant difference regarding age between the two groups (Table 2). However, more pediatric dentists (61.0%) were employed in public service compared to other specialists (Table 2), and 82.9% of pediatric dentists provided dental care (emergency or routine) during lockdown (Table 3). These differences were statistically significant.

The further analysis presented as supplementary material revealed no statistically significant differences between pediatric dentists’ and other specialists’ compliance to using precaution measures or PPE during dental treatment. Table S1 shows the adherence to universal preventive measures and PPE use. More than half of all other specialists and pediatric dentists involved in the study used the following recommended universal precautionary measures: phone triage (72.0% and 61.5%, respectively); reducing appointments (74.6% and 65.9%, respectively); elective treatment for vulnerable patient groups (62.3% and 54.9, respectively); handle disinfection (83.3% and 76.9%, respectively); disinfection of surfaces (70.6% and 60.4%, respectively); health assessment before treatment (75.4% and 71.4%, respectively); sanitizing hands of patients in dental office (82.5% and 74.7%, respectively); obligatory masks for patients (80.2% and 56.9%, respectively); frequent waiting room ventilation (83.3% and 70.3%, respectively); removing unnecessary items from the waiting room, such as magazines, toys, etc. (61.5% and 53.8%, respectively); 10 min-treatment room ventilation between patients (54.0% and 54.9%, respectively); 70% ethyl alcohol surface disinfection (79.4% and 79.1%, respectively); and obligatory washing of dentist’s hands (88.1% and 87.9%, respectively). Not using aerosol generating procedures (AGP) was more frequent in pediatric dental offices (36.3%) compared to other specialists’ offices (34.5%); however, no statistically significant difference was found. Most specialists (both other specialists and pediatric dentists) used surgical masks (83.3% and 83.9%, respectively), while using respirator masks was reported in one-third of participants (37.9% and 31.0%, respectively). Moreover, most dentists used visors (86.1% and 92.0%, respectively), while googles were used by only half of participants (53.8% and 56.3%). Pediatric dentists, in general, showed slightly lower adherence to using precaution measures and PPE compared to other specialists. However, wearing masks for patients was significantly less frequently mandatory in pediatric dental waiting rooms (65.9%, *n* = 60) compared to adult dental offices (80.2%, *n* = 202) (Pearson’s chi-square, *χ^2^ =* 7.5 (*p* < 0.01)). In addition, adult specialists statistically significantly more often used frequent waiting room ventilation (Pearson’s chi-square, *χ^2^ =* 7.0 (*p* < 0.01)) and preoperative mouth rinse with the 0.12 to 0.2% solution of chlorhexidine (Pearson’s chi-square, *χ^2^ =* 3.9 (*p* < 0.05)).

None of the pediatric dentists involved in the study complained about PPE unavailability compared to other specialists (11.5%) (Table 4).

However, almost one-quarter of all specialists—both pediatric dentists and others (26.2%)—stated that they needed clear, step-by-step professional guidance in a national language to provide safe clinical activities. Similarly, like other specialists (*n* = 52, 10.9%), only 9.8% (*n* = 16) of pediatric dentists stated that they attended any training on COVID-19. However, when only pediatric dentists were analyzed in detail, the results revealed that 11.2% (*n* = 13) of pediatric dentists working in the public sector attended education on COVID-19, while only one pediatric dentist working in a private dental office acquired the same knowledge. On the other side, a high frequency of specialists—both pediatric dentists (*n* = 99, 60.4%) and other specialists (*n* = 229, 47.8%)—responded that they need more knowledge on COVID-19.

Although there was no statistically significant difference, the results presented in Table 5 revealed that pediatric dentists were significantly more frequently tested and almost twice as likely to test positive for COVID-19 (3.0%) compared to other specialists (1.9%). Further analysis revealed that twice as many pediatric dentists experienced at least one of the specific or nonspecific COVID-19 symptoms during the state of the emergency in their country (13.4% in pediatric dentists vs. 6.1% in other specialists, OR = 1.89, 95%CI: 1.038–3.454). More detailed investigation revealed that out of *n* = 112 public sector pediatric dentists, 3.4% (*n* = 4) were diagnosed with COVID-19, while none of the private sector pediatric dentists tested positive nor were diagnosed with SARS-CoV-2 infection (Pearson’s chi-square, *χ^2^ =* 0.9, *p* = 0.32). Additionally, although not statistically significant (Pearson’s chi-square, *χ^2^ =* 1.5, *p* = 0.21), more pediatric dentists from the public sector (16.4%, *n* = 19) presented with COVID-19-like symptoms compared to pediatric dentists from private practice (7.1%, *n* = 2).

## 4. Discussion

Pediatric dentists showed a twice as high occurrence of specific and nonspecific COVID-19 symptoms and COVID-19-positive tests compared to other dental specialists during the first wave of the pandemic in the spring of 2020. Most pediatric dentists (82.9%) provided dental treatment during the state of the emergency. Pediatric dentists were, in general, slightly less compliant to using precaution measures or PPE during dental treatment than other specialists.

According to the authors’ knowledge, this is the first study to analyze pediatric dental service and the risks of COVID-19 infection in pediatric dentists during the pandemic. Furthermore, it highlighted that pediatric dental service mainly was available during the state of emergency via public health institutions (82.9% of pediatric dentists involved in the study provided emergency and routine dental treatment). There is a need to raise awareness on the constant need to protect both patients and oral health care staff even when patients might be asymptomatic. It was established that droplets, saliva, and aerosols are the main routes for transmission of SARS-CoV-2 [14], which puts the dentists and healthcare workers at high risk of being infected and even transmitting the disease to their families or other patients [15]. Keeping a physical distance is impossible during dental treatment and aerosol-generating procedures represent the core of modern dentistry; the risks for spreading the virus in the dental office are considered high.

The COVID-19 pandemic changed the way people lived globally, endangered human lives, and damaged global health, increasing health inequalities and involving oral health issues [6]. Due to lockdowns and curfews, elective dental procedures have been postponed, hence contributing to neglecting oral health. However, according to our results, 82.9% of pediatric dentists provided routine or urgent dental treatment during lockdown, mostly in public health centers.

During the first couple of months of the pandemic in 2020, the scientific community was primarily focused on easing the burden imposed on the health care system and hospital workers, neglecting dental professionals [16]. National and international dental associations and the scientific community soon provided dental recommendations, guidelines (ADA, CDC, IADH), and educational opportunities via online platforms. The rapid pace of news and uncertainties around the viral genesis, transmissibility, and pathogenesis led to information voids that were conveniently and quickly filled with a huge amount of social media posts in a new phenomenon known as “infodemic” [17,18,19,20]. The rapid spread of information, some of which were later discredited, has floundered people and created widespread anxiety [21]. Keeping in mind that there is a huge variety of epidemiological circumstances across the world, including different national laws, regulations, levels of the community transmission, phase of the pandemic. and health system responsiveness, it would be impossible to provide unique and joint recommendations. It is possible that dentists had difficulties in finding reliable information regarding recommended preventive measures and their efficiency, especially in their mother tongue. Therefore, Southeastern oral health professionals, especially those without good English language proficiency, web browsing, and international networking skills, who worked clinical hours during lockdown periods were forced to rely on their clinical judgement depending on the availability of PPE and precaution measures.

This study revealed that pediatric dentists showed lower compliance to using PPE than other specialists, the main differences being noticed in wearing a mask in the waiting room and using mouth rinses before starting the treatment. Wearing protective masks in public for children under the age of five was not obligatory during the first wave of the pandemic and is still not recommended [22]. WHO recommends mask-wearing in children older than 12 and in the 5–11 age group depending on specific risks. Wearing masks is not recommended for those younger than five years old [22]. Our results might suggest updating recommendations for pediatric dental institutions even when patients are younger than 12 or younger than five years old. The Centers for Disease Control and Prevention has updated (9 July 2021) its guidance to recommend that students, teachers, staff and visitors wear masks indoors regardless of vaccination status as well as wearing masks indoors by all individuals (age two and older) [23]. This explains our results revealing significantly lower compliance of patients to wear masks in the waiting room of pediatric dentists’ offices vs, other practices during the first wave of the pandemic. Additionally, it would be unacceptable to use specific anti-microbial mouthwash (due to its bad taste) in children before treatment due to characteristics of children as dental patients. The use of PPE and protective glasses, face shields, masks, gloves, shoe covers, and head caps in the routine dental practice has become mandatory to protect the operator from contamination with blood and saliva while treating patients because asymptomatic patients can be potentially contagious. However, using additional PPE in the pediatric dental office calls for additional behavioral management techniques considering the changed environment in the dental office and dental team physical appearance [24].

Our results revealed that all pediatric dentists with a positive test were from the public sector. In addition, twice as many pediatric dentists with COVID-19 symptoms were from the public sector (16.4% vs. 7.1%). Higher patient load in public health institutions might bring higher transmission risk. Therefore, pediatric dental professionals need to find the best way to minimize the risk for their health and for spreading the infection while providing dental treatment to the patient.

In our study, the frequency of COVID-19-positive tests among dentists and pediatric dentists was 1.9% and 3.0%. Other dental surveys showed that despite the potential high risk for the infection, dentists showed amazingly low rates of COVID-19 in 2020: 0.8% in China [25], 0.9% in USA [26], 10% in Spain [27], and 10.8% in Lombardy in Italy [28]. Despite potential risks, dentists’ long tradition in using PPE and infection prevention was rewarded. Even more, it is considered that water or air spray and high suction that is usually used in dentistry during AGP reduce viral load in dental aerosols [29]. Considering the lower incidence of COVID-19 and its community transmission in Southeastern European countries during March and April 2020, the identified relatively high prevalence could be attributed to the lack of adequate knowledge and/or resources. Both dental health professionals and dental undergraduates need to acquire enough knowledge because of their role in treating their patients safely. An international multicenter study involving dental academics around the world showed that having a PhD, a larger social network, and a higher patient load increased education on the mode of transmission, diagnosis, and preventive dental practices [30]. Evidence on SARS-CoV-2 transmission through the aerosols and droplets during dental treatment is lacking, but it has been presumed that these aerosols are equal to those during medical interventions [29]. Caution in the dental office is necessary due to possibility to treating a patient with mild symptoms or without any symptoms. Moreover, more research is necessary in order to design strong recommendations that are adapted to pediatric dental practice settings and in accordance with behavioral principles. We believe that further research is necessary to find the best possible way to educate dental professionals regarding COVID-19. Keeping in mind that dental academics are skilled in using data for research and obtaining information via various educational channels, they are more willing to accept new ideas and knowledge, so they could be targeted as potential educators who could share knowledge in their mother tongue to non-academic dentists [30]. As revealed and confirmed in this study, National Dental Chamber resources might be used as very convenient method for delivering scientifically proven information.

This study has brought to light some interesting findings in the specific setting of Southeast Europe. During the last couple of decades, all countries in the Balkan region experienced financial and health care challenges due to transitional crisis [31]—similar health system models showed weaknesses due to financial losses, so poor availability of PPE was expected. However, even high-resource countries struggled with PPE availability during this public health crisis [32]. Maybe our setting presented in this survey might be one of the excellent examples for PPE-optimization strategies in low-recourse settings since PPE was targeted to the public health centers (mostly public pediatric dental institutions) that were available during the lockdown. As presented in Table 3, our results confirmed that one-third of specialists stopped clinical practice mostly due to obligatory national epidemiological restrictions (Table 4); yet, pediatric dentists mostly performed clinical activities, and unavailability of PPE was not observed in this group (0% pediatric dentists vs. 11.5% other specialists).

One of the strengths of this study is that participants were reached in each country via adequate platforms to avoid duplicate answers, and the invitations were sent through the national Dental Board systems, guaranteeing good coverage and a potential to obtain a representative pool of answers. The system ensured anonymous answers, so dentists were assured that no identification was possible. Although this survey provides valuable and interesting information, caution is needed when interpreting results due to some limitations. Our study had sufficient power to estimate the prevalence of SARS-CoV-2-positive pediatric dentists in the target region but was underpowered to detect significant differences between general and pediatric dentists. In addition, pediatric dentists from countries such as Montenegro, North Macedonia, Greece, and Cyprus were underrepresented in our study. Additionally, our results could not deduce causes and reasons for dentists’ infections. The design of our questionnaire did not involve questions about risks of infection transmission through the community (travels, personal contact, etc). This was an observational study; the results were obtained from the questionnaire, so all data regarding symptoms are self-reported. Considering additional waves of COVID-19 in most countries and these limitations, a follow-up study might be able to build on these results and answer some of the open questions.

## 5. Conclusions

Considering that most pediatric dentists provided dental treatment during lockdown in their countries in public health centers, and they will continue to work during pandemic, it is important to ensure a safe environment and continuous dental care. According to our results, we observed that special attention and further research should focus on finding the best possible way to promote and adapt using preventive and protective measures and PPE in pediatric dental settings to be behaviorally acceptable for pediatric patients. In addition, more efforts should invest in dental education regarding COVID-19, so clear and scientifically proven information can be widely available in the mother tongue. More similar research is needed to confirm these results in a larger sample of pediatric dentists. Our results suggest that pediatric dentists might be at higher risk for COVID-19 infection.

## Figures and Tables

**Table 1:** Main survey overview.

	Survey Period	Lockdown Period	Number of Registered Dentists	Number of Registered Pediatric Dentists	Number of Reached Dentists	Number of Reached Specialists	Number of Reached Pediatric Dentists
Albania	17.07–31.07	12.03–11.05	2776	21	153 (5.5%)	23	4
Bosnia and Herzegovina	29.06–14.07	11.03–29.05	2250	92	213 (9.54%)	76	25
Montenegro	17.07–31.07	18.03–04.05	591	43	110 (18.9%)	34	6
North Macedonia	17.07–31.07	30.03–13.05	2800	76	24 (0.9%)	6	0
Serbia	17.07–31.07	15.03–07.05	4677	360	1460 (31.2%)	483	129
Cyprus	17.07–31.07	16.03–4.05	1017	0 *	176 (17.3%)	16	0
Greece	17.07–31.07	16.03–4.05	10,200	0 *	45 (0.44%)	5	0
Total			24,311	592	2181 (8.9)	643	164

* Cyprus and Greece do not have an officially recognized specialization in pediatric dentistry, but general dentists (or master’s degree) that occupy with children.

**Table 2:** Sample summary.

	Other Specialists *479 (100%)*	Pediatric Dentists *164 (100%)*	All Specialists *643 (100%)*	*p*-Value
**Gender**				0.04
Male	54 (11.3%)	6 (3.7%)	60 (9.3%)	
Female	425 (88.7%)	158 (96.3%)	583 (90.7%)	
Pearson’s Chi-Square, *χ^2^* = 8.4				
Age (years) (mean age ± SD)	47.3 (±9.7)	48.1 (±7.6)	47.5 (±9.2)	0.38
*t*-test for equality of means, *t* = −0.878				
**Type of practice**				
Private Practice Owner	262 (54.7%)	20 (12.2%)	282 (43.9%)	<0.01
Private Practice Employee	63 (13.2%)	8 (4.9%)	71 (11.0%)	
Public Service	78 (16.3%)	100 (61.0%)	178 (27.7%)	
Both Private and Public Service	40 (8.4%)	20 (12.2%)	60 (9.3%)	
Research	36 (7.5%)	16 (9.8%)	52 (8.1%)	
Pearson’s Chi-Square, *χ^2^* = 135.3				

**Table 3:** Working regime during the state of emergency in pediatric dentistry compared to other specialties.

	Other Specialists *n* (%)	Pediatric Dentists *n* (%)	Total *n* (%)
Stopped all clinical activities	68 (14.2%)	13 (7.9%)	81 (12.6%)
Stopped all clinical activities and provided advice/triage via telephone	127 (26.5%)	15 (9.1%)	142 (22.1%)
Limited clinical activity to emergency dental care	219 (45.7%)	106 (64.6%)	325 (50.5%)
Provided routine dental treatment	65 (13.6%)	30 (18.3%)	95 (14.8%)
Total	479 (100.0%)	164 (100.0%)	643 (100.0%)
(Pearson’s Chi-Square, χ^2^ = 30.9, *p* < 0.01)			

**Table 4:** Reasons for stopping the clinical practice during first wave of COVID-19 pandemic.

	Other Specialists	Pediatric Dentists	Total
	*n* (%)	*n* (%)	*n* (%)
It was obligatory	52 (31.5)	5 (22.7)	57 (30.5)
Absence of guidelines	43 (26.1)	6 (27.3)	49 (26.2)
Unavailability of PPE	19 (11.5)	0 (0)	19 (10.2)
Personal reasons	51 (32.0)	11 (50.0)	62 (33.2)
Total	165 (100.0)	22 (100.0)	187 (100.0)

χ^2^_(3)_ = 11.1, *p* = 0.160.

**Table 5:** Prevalence of COVID-19 testing and presence of one or more specific or nonspecific COVID-19 symptoms in dentists during first wave of COVID-19 pandemic.

	Other Specialists	Pediatric Dentists	Pearson’s Chi-Square Value
	*n* (%)	*n* (%)	(*p*-Value)
Presence of symptoms	29 (6.1)	22 (13.4)	*χ^2^ =* 9.1 (*p* < 0.01)
COVID-19 testing	106 (22.1)	56 (34.1)	*χ^2^ =* 9.4 (*p* < 0.01)
Positive tests	9 (1.9)	5 (3.0)	*χ^2^ =* 0.8 (*p* = 0.4)

## Data Availability

The data are available upon reasonable request to the first or corresponding author.

## Supplementary Materials

The following are available online at https://www.mdpi.com/article/10.3390/ijerph182211795/s1, Table S1: Attendance to universal precaution measures and PPE use.
