# Supplementary material for: Pediatric Dentists’ Service Provisions in South-East Europe during the First Wave of COVID-19 Epidemic: Lessons Learned about Preventive Measures and Personal Protective Equipment Use
Source: Int J Environ Res Public Health. 2021 Nov 10;18(22):11795. doi: 10.3390/ijerph182211795 (PMC8622949; doi:10.3390/ijerph182211795)
Supplement: Supplementary file 1 [file ijerph-18-11795-s001.zip › ijerph-1423262-supplementary.pdf]

International Journal of
Environmental Research and Public Health

MDPI

Supplementary Table

Table S1. Adherence to universal precaution measures and PPE use.

Universal precaution measures	Other specialists n (%)	Pediatric dentists n (%)	All specialists n (%)	p value
Phone Triage	179 (71.0)	56 (61.5)	235 (68.5)	0.095
Appointments reduced to avoid overcrowding of the waiting room	188 (74.6)	60 (65.9)	248 (72.3)	0.113
Postponement of routine treatment for elderly or vulnerable patients	157 (62.3)	50 (54.9)	207 (60.3)	0.219
Handle disinfection several times a day	210 (83.3)	70 (76.9)	280 (81.6)	0.176
Disinfection of push buttons and chairs several times a day	178 (70.6)	55 (60.4)	233 (67.9)	0.074
Assessment of patients' health on/before arrival	190 (75.4)	65 (71.4)	255 (74.3)	0.458
Measuring patients' body temperature	91 (36.1)	29 (31.9)	120 (35.0)	0.467
Measuring body temperature of all dental team members	56 (22.2)	15 (16.5)	71 (20.7)	0.247
Obligatory sanitizing patients' hands	208 (82.5)	68 (74.7)	276 (80.5)	0.107
Phisical distance in the waiting room	142 (56.3)	45 (49.5)	187 (54.5)	0.257
Patients waiting outside the office until called in	124 (49.2)	38 (41.8)	162 (47.2)	0.223
Patients wearing masks in the waiting room	202 (80.2)	60 (56.9)	262 (76.4)	0.006
Frequent waiting room ventilation	210 (83.3)	64 (70.3)	274 (79.9)	0.008
Removal of magazines, toys, and books from the waiting area	155 (61.5)	49 (53.8)	204 (59.5)	0.202
Storage of coats, bags and other items outside the treatment room	66 (26.2)	15 (16.5)	81 (23.6)	0.062
Rinsing with 1% hydrogen peroxide mouthwash before treatment	148 (58.7)	45 (49.5)	193 (56.3)	0.126
Rinsing with chlorhexidine 0.12-0.2% mouthwash before treatment	33 (31.1)	5 (5.5)	38 (11.1)	0.048
Rinsing with 0.2-1% iodopovidone mouthwash before treatment	27 (10.7)	8 (8.8)	35 (10.2)	0.603
Rinsing with alcohol and essential oils mouthwash before treatment	8 (3.2)	2 (2.2)	10 (2.9)	0.635
Rinsing with 0.05-0.10% Cetylpyridinium chloride mouthwash before treatment	4 (1.6)	2 (2.2)	6 (1.7)	0.703
Rinsing with commercial mouthwash	20 (7.9)	6 (6.6)	26 (7.6)	0.678
Ventilation of the treatment room for at least 10 minutes after each patient	136 (54.0)	50 (54.9)	186 (54.2)	0.873
Surface disinfection with 70% ethyl alcohol	200 (79.4)	72 (79.1)	272 (79.3)	0.961
Surface disinfection with 0.5% sodium hypochlorite	96 (38.1)	24 (26.4)	120 (35.0)	0.044
Surface disinfection using disinfectants with other active ingredients	88 (34.9)	26 (28.6)	114 (33.2)	0.270
Obligatory washing dentist's hands before and after each procedure	222 (88.1)	80 (87.9)	302 (88.0)	0.963
Disposal of all single used protective devices and disinfection of non-disposable devices	197 (78.2)	66 (72.5)	263 (76.7)	0.275
Not using AGP such as high-speed dental handpiece or ultrasonic instruments	87 (34.5)	33 (36.3)	120 (35.0)	0.765
Using aerosol generating procedures such as high-speed dental handpiece or ultrasonic instruments in separated well ventilated room, at the end of shift with respirator mask on	66 (26.2)	17 (18.7)	83 (24.2)	0.152
**Personal protective equipment**				
Surgical mask	209 (83.3)	73 (83.9)	282 (83.4)	0.890

Table S1. Adherence to universal precaution measures and PPE use.

Universal precaution measures	Other specialists n (%)	Pediatric dentists n (%)	All specialists n (%)	p value
Resiprator mask	95 (37.9)	27 (31.0)	122 (36.1)	0.233
Visor	216 (86.1)	80 (92.0)	296 (87.6)	0.151
Breathable disposable gown/coverall	64 (25.5)	31 (35.6)	95 (28.1)	0.070
Water repwllent disposable gown/coverall	33 (13.1)	12 (13.8)	45 (13.3)	0.879
Googles	135 (53.8)	49 (56.3)	184 (54.4)	0.682
Sterile gloves	102 (40.6)	25 (28.7)	127 (37.6)	0.048
Non-sterile gloves	178 (70.9)	65 (74.7)	243 (71.9)	0.497
Rotating instrument with anti-retraction valve	13 (5.2)	4 (4.6)	17 (5.0)	0.831
Rubber dam	16 (6.4)	2 (2.3)	18 (5.3)	0.145
Reusing or prolonged using of PPE	47 (18.7)	20 (23.0)	67 (19.8)	0.390
Using disposable caps only single use	105 (41.8)	30 (34.5)	135 (39.9)	0.228
Using disposabel gowns single use	66 (26.3)	15 (17.2)	81 (24.0)	0.088
